# N450 and LPC Event-Related Potential Correlates of an Emotional Stroop Task with Words Differing in Valence and Emotional Origin

**DOI:** 10.3389/fpsyg.2017.00880

**Published:** 2017-05-30

**Authors:** Kamil K. Imbir, Tomasz Spustek, Joanna Duda, Gabriela Bernatowicz, Jarosław Żygierewicz

**Affiliations:** ^1^Faculty of Psychology, University of WarsawWarsaw, Poland; ^2^Faculty of Physics, University of WarsawWarsaw, Poland

**Keywords:** duality of emotions, emotional stroop task, mechanisms of cognitive control, ERP, emotional words

## Abstract

Affective meaning of verbal stimuli was found to influence cognitive control as expressed in the Emotional Stroop Task (EST). Behavioral studies have shown that factors such as valence, arousal, and emotional origin of reaction to stimuli associated with words can lead to lengthening of reaction latencies in EST. Moreover, electrophysiological studies have revealed that affective meaning altered amplitude of some components of evoked potentials recorded during EST, and that this alteration correlated with the performance in EST. The emotional origin was defined as processing based on automatic vs. reflective mechanisms, that underlines formation of emotional reactions to words. The aim of the current study was to investigate, within the framework of EST, correlates of processing of words differing in valence and origin levels, but matched in arousal, concreteness, frequency of appearance and length. We found no behavioral differences in response latencies. When controlling for origin, we found no effects of valence. We found the effect of origin on ERP in two time windows: 290–570 and 570–800 ms. The earlier effect can be attributed to cognitive control while the latter is rather the manifestation of explicit processing of words. In each case, reflective originated stimuli evoked more positive amplitudes compared to automatic originated words.

## Emotional stroop task

The Emotional Stroop Task (EST) is a modification of the standard procedure introduced by Stroop (1935), which allows measuring the cognitive control in the case of interference control (Nigg, 2000). Interference in EST is made by the affective content of a word and arises as a result of the competition of two processes (Imbir, 2016a). The first process is an automated reading and understanding of the semantic meaning of words. This captures participants' attention and generates slowdown in the second, controlled process. The second process is related to the task of naming of the font color. In the classical Stroop Test, the interference is caused by the understanding the meaning of words, which are the names of colors. The interference is observed when comparing congruent (e.g., responding “red” to a word “RED” written in red font) and incongruent (responding “red” to word “BLUE” written in red font) trials. The congruent trials are perceived as easier and are performed faster. The EST differs from the classical Stroop Task in the nature of the interference measured (Nigg, 2000; Larsen et al., 2006). In the EST, an incongruent and congruent trials are constructed by carefully choosing words so that they differ only in one affective factor (e.g., valence or arousal), while they are matched in respect of other properties (e.g., frequency, grammatical class, or length). This allows drawing an unambiguous conclusion from collected data. As the incongruent trials, involving the automatic disposition of attention toward a task-irrelevant lure (supposed to be analogical to the classical Strop effect) are treated those with extreme levels of the chosen factor, while congruent are those with moderate or neutral levels of the factor. For example, as far as valence is considered in the EST, trials with positive or negative words are considered incongruent while trials with neutral words are considered congruent (Burt, 2002; Imbir and Jarymowicz, 2013).

### Factors underlying behavioral effects in EST

The behavioral phenomenon of EST has been discovered in clinical trials. Subjects experiencing particular trauma had longer reaction times for trauma-related words than for other words (Watts et al., 1986; McKenna and Sharma, 1995, 2004). The EST effects were demonstrated also in a clinical and subclinical psychological probes suffering from anxiety disorders (see Williams et al., 1996 for a review). The EST appeared to be a useful tool to detect the source of anxiety, because longer reaction times were observed for words connected in meaning with a particular source of threats. Subsequently, it was reported that EST slowdown could be observed in a normal population with no trauma experience (Nigg, 2000; Larsen et al., 2006; Siakaluk et al., 2014). Valence was shown to influence reaction latencies in cases of words with negative valence (e.g., Williams et al., 1996; McKenna and Sharma, 2004) and also with positive valence (e.g., Pratto and John, 1991; Richards et al., 1992; McKenna and Sharma, 1995). The effect for positive words was usually smaller than for negative words. Also, individual experience with objects or states represented by the words was shown to boost the slowdown (Reiman and McNally, 1995).

Careful inspection of valence effects revealed that other factors, not controlled in advance, could explain the behavioral effects (Burt, 2002; Larsen et al., 2006). Those factors, identified as far, were arousal load and frequency of appearance in language. It appeared that arousal causes slowdown that is independent from valence (Dresler et al., 2009; Imbir, 2016a). High arousing words result in a greater slowdown in reaction latencies than low arousing stimuli. Also less frequent words cause higher slowdown than more frequent ones (Burt, 2002). Some recent results suggest that origin of an affective state may be an another factor responsible for slowdown observed in EST (Imbir and Jarymowicz, 2013), thus the question concerning factors underlying EST performance is still open. Since the valence effect was shown to be confusing and appeared to be blurred by other factors effects, we decided to ask a question concerning the nature of emotion and its' influence on EST. To find the answer, we have applied the dual-mind theories perspective, especially recently introduced framework concerning emotion and cognition interactions viewed from dual mind perspective (Imbir, 2016b).

### Duality of mind and EST

Recently, the duality of emotion framework was proposed in order to explain diversity of emotions (Jarymowicz and Imbir, 2015) as well as emotion-cognition interactions (Imbir, 2016b). This proposition is based on duality of mind theories (for broad review see Gawronski and Creighton, 2013), distinguishing between so-called automatic and controlled processes. There is a huge diversity of dual-mind theories, but all of them highlight the main role of the above-mentioned processing modes. Taking into account Epstein's (2003) proposition of existence of two aspects of mind, namely experiential and rational, we argue that both have their cognitive (c.f. Strack and Deutsch, 2004, 2014; Kahneman, 2011) manifestations in form of associative (or heuristic) vs. systematic (or rational) processes and emotional (Jarymowicz and Imbir, 2015) manifestations in form of automatic vs. reflective originated emotional states (c.f. Imbir, 2016b). In the classical view on emotions seen from the dual-mind theory framework, they were thought to be associated only with simplified processing of experiential mind (c.f. Epstein, 2003; Kahneman, 2011), but such view was not sufficient to describe more complex, self-conscious emotions (Weiner, 2005). The emotion duality model (Jarymowicz and Imbir, 2015) states that emotional experiences themselves can originate due to either automatic or reflective evoking mechanisms. This implicates, that affective processes are not necessarily automatic and may be understand as a results of controlled and rational mind processing (c.f. Reykowski, 1989; Strack and Deutsch, 2004, 2014). What is more, according to the classical theories only one of the mind-system could be tested in a given experimental protocol with use of a single task, attention manipulation or stimulus presentation parameters, while the emotional duality model applied to emotional words processing allows to study activation of either of the systems within the same experimental protocol, for the same subject, by treating the ability of evoking automatic or controlled mind processes as inherent property of individual word meanings. This fact address the most important critique addressed to dual-mind perspective (c.f. Ferguson et al., 2014).

In the case of experiential mind, so-called automatic originated emotions are characteristic for the direct affective responses to environmental stimulation. Such responses do not need language to appear and we assume that they are based on evaluation of criteria of biological value (Damasio, 2010). Certain objects help in maintaining life and thus are automatically evaluated as pleasant (e.g., fatty and sweet foods), while other things are threats to life and thus are evaluated as unpleasant (e.g., smelly or sour meals). The experiences of automatic originated emotions can be labeled with words (e.g., pain) and thus are widely represented in language (Rolls, 2000). In the case of rational mind, so-called reflective originated emotions are postulated (Jarymowicz and Imbir, 2015). Their most characteristic feature is that formation of reflective originated emotional states requires language (Reykowski, 1989; Strack and Deutsch, 2004, 2014). The propositional mechanisms in the form of evaluative standards (Reykowski, 1989) serve as a source of reflective emotions. Reflective emotion arises when a situation or behavior is compared to a standard represented in the mind. It is clear that the evaluative standards are both subject dependent and plastic; thus different reflective emotions toward single situation can arise in different subjects, or even in the same subject at different times (Jarymowicz and Imbir, 2015).

**Figure 1:**
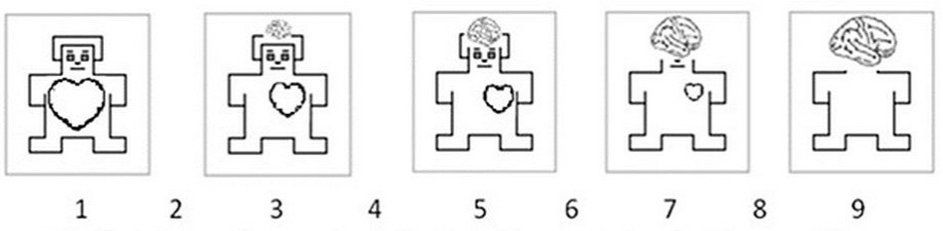
**Self-Assessment Manikin (SAM) scale and its description developed to measure origin dimension in Affective Norms for Polish Words study (Imbir, 2015, 2016c)**. SAM scale was accompanied by a description of its' meaning: “The first picture shows an individual who is overwhelmed with appeals from the heart—words that could represent these experiences include being beside oneself, complete commitment, full engagement, impulsivity, spontaneity, lack of hesitation. The last picture shows a person who is under the sway of the mind, who is reflective—words that could be used to represent this state include feelings that result from contemplation, planning, consideration, prediction, choices, or comparisons.”

To measure the nature of affective reaction (automatic or reflective), the origin dimension was proposed and used in the case of assessing affective reactions to words (Imbir, 2015, 2016b). On the theoretical level (c.f. Epstein, 2003; Strack and Deutsch, 2014; Jarymowicz and Imbir, 2015; Imbir, 2016b) emotional origin is a clearly dichotomic factor, but the direct measurement of the processing style underlying emotion formation is yet not possible, thus we have to base on subjective perception toward the processing mechanisms. Origin dimension is measured on a scale constructed as a type of Self-Assessment Manikin (SAM) scale (Lang, 1980). This scale allows for non-verbal assessments of feelings connected with presented stimuli (c.f. Figure 1). SAM scale was supplemented with a description of its meaning in order to provide unambiguous interpretation of the “origin” concept. We think that origin is not an intuitive dichotomy when emotions are considered (Jarymowicz and Imbir, 2015). Origin is rather a hidden underlying mechanism (Russell, 2003); thus we use in creation of SAM scale the heart vs. mind metaphor (c.f. Figure 1 legend), widely represented in culture, as a good exemplification of dual-mind dichotomy of underlying processes (Imbir, 2015). Heart represents immediate reactions that do not require hesitation, in contrast to mind that represents careful inspection of all opportunities and interpretations of the situation. We argue that some of the emotions are based on non-verbalized criteria of evaluation, as proposed by Damasio's (2010) biological value. Other emotions require cognitive resources and language to interpret and appraise reality. Mechanisms underlying those more cognitive-based emotions are evaluative standards (Reykowski, 1989) or propositional thinking (Strack and Deutsch, 2004) based on processing with the use of sentences and rules of logic. The first case describes so-called automatic emotions and the second case reflective ones. Origin SAM scale allowed for reliable measurement of perception of automatic vs. reflective origins of affective reactions to words (Imbir, 2015, 2016c). Nevertheless, it appeared that not all stimuli had unambiguous associations of their origins. In real world most of states are results of activation of both mental systems (Epstein, 2003; Kahneman, 2011; Jarymowicz and Imbir, 2015). Also this aspect was found in the SAM scale measures collected. Some words received moderate assessments (based on ambiguous interpretations made by different people), thus in fact not allowing for specification of certain origin. We treat those words as stimuli with no specified origins, because no particular and clear associations were drawn (cf. Imbir et al., 2016). Distinct mechanisms underlying affective processes formation, that are reflected in words connotations defined from a dual-mind perspective, can be compared in a single experiment, due to high level of similarity for materials specific to both mind systems (Imbir, 2017). In a traditional view on dual-mind systems, there was an expectation to operationalize them as a distinct, especially because experiential system works in visual representations (Epstein, 2003), while reflective systems is based on verbalizations (Strack and Deutsch, 2004). The lack of possibility to create a single experiment testing consequences of both mind systems was the main weakness raised for dual-mind perspective (Ferguson et al., 2014). Origin factor, found to be reliably measured for words (Imbir, 2016c), offers an important advantage for understanding the role of dual mind processes in word processing and understanding of affect influence on cognitive control. Figure 1 presents the SAM scale for origin assessments.

EST is a type of task that involves the two types of processes: automated and controlled (Imbir, 2016a). Explicit task is the controlled one. It is not a standard action to ignore the meaning of a word and focus on its font color instead. Processing of this action requires an effort (Kahneman, 2011) in order to prevent (Nigg, 2000) more automated reading of words and subsequent understanding of their semantic meaning (Imbir, 2016a). The current study tests a hypothesis, posted in dual-mind model of emotion-cognition interactions (Imbir, 2016b), stating that cognitive and emotional processes are in fact results of broader mental systems (experiential and rational). If it is true (c.f. Imbir, 2017), triggering automatic emotion should activate experiential mind processing (automatic one), while reflective emotions should activate rational mind mechanisms (controlled ones). Taking into account the dual nature of EST, one may conclude that processing in this type of task should be specific to the nature of stimuli presented, stimulating one or another system, thus influencing the pool of resources available for completion of the task (c.f. Imbir, 2016a; p. 4, Figure 2). Automatic originated stimuli, as associated with experiential mind, should activate or enhance automated part of EST, thus enlarge reaction latencies. Simply automatic originated stimuli should make reflex of reading stronger, because decoding of those stimuli triggers the experimental mind responsible for automated actions. Opposite effects should be observed for reflective originated stimuli. They should activate controlled part of EST, as they are associated with rational mind governing controlled processing. Understanding of the reflective originated stimuli meaning should trigger rational mind and thus controlled processing should be stronger.

**Figure 2:**
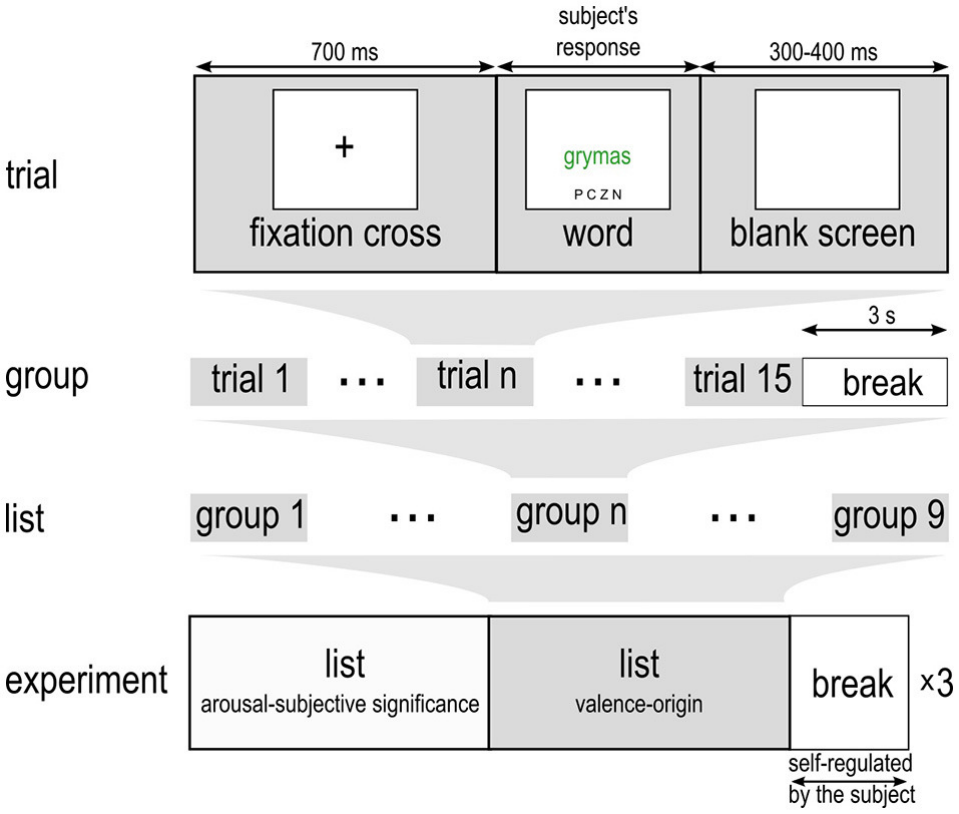
**Outline of the experimental procedure**.

Guided by described above expectations, a behavioral results of early EST study, involving words that differed in levels of origin and valence (Imbir and Jarymowicz, 2013), showed that valence effects disappear when stimuli are controlled for origin (contrasted orthogonally with valence). Slowdown in reaction latencies was observed when automatic originated words were used in EST, but it was not observed when neutral or reflective words were considered. It is important to highlight the fact that words used in the Imbir and Jarymowicz (2013) study were selected by competent judges (assessing compliance with automatic and reflective systems of evaluation definitions) and thus were explicitly connected with both investigated origins (automatic vs. reflective). Words from Imbir and Jarymowicz (2013) study were contrasted by valence and origin levels and matched with respect to their frequency, but were not matched with arousal. Therefore, we decided to probe the effects with a new set of words, carefully chosen from assessments performed in normative studies for words (Imbir, 2015, 2016b).

Using Russell's (2003) concept of bimodal affective space, composed of two orthogonal dimensions of (1) valence, representing pleasantness vs. unpleasantness of an affective state, and (2) arousal, representing the activation underlying an affective state, we decided to check each dimension in the context of duality of emotion framework. In dual mind emotion-cognition relation framework (Imbir, 2016b), there is a postulate of existence of a mind system specific aspects of both dimensions, namely origin of an affective state for valence and subjective significance for arousal. Taking into account origin of an affective state, we treat it as a process that influences affective processing but should be thought of as outside of valence and arousal affective space. Defined by Russell (2003) valence and arousal are the simplest conscious accessible affective feelings. Origin is not an intuitive factor and as mentioned earlier, the measurement of origin was not unambiguous (c.f. Materials and Methods Section). Apart this, both automatic and reflective originated states should possess two distinct valences, as well as they should differ one another by the levels of arousal. Origin reflect the basic distinction of two types of processing (based on automatic vs. controlled mechanisms) in the domain of emotion formation, while valence and arousal affective space define subjectively perceived pleasantness and activation. As far as the activational aspect of an affective reaction is considered, we distinguish between arousal-like activation, which is specific to the experiential system (Epstein, 2003), and a postulated subjective significance (Imbir, 2015), which is an activation for reflective mind processes (Imbir, 2016b). It was shown (c.f. Imbir, 2016a) that subjective significance shaped the arousal effect—the slowdown of reaction latencies observed in EST—in such a way that latencies were reduced for both low and high subjectively significant and high arousing stimuli compared to medium subjectively significant and high arousing stimuli. An analog of this pattern of behavioral results was observed in an electrophysiological (EEG) study focusing on Event-Related Potential (ERP) correlates of processing both dimensions in EST (Imbir et al., submitted, second list described in Procedure Section). Although comparable manipulation of origin and valence did not reveal behavioral effects (unpublished research report), we decided to check if the ERP measurements would uncover effects of manipulation of origin and valence factors, since ERP measurements are more sensitive and are able to show underlying processes even when the behavioral level of analysis shows no differences (Thomas et al., 2007).

### ERP correlates of word processing and EST performance

Studies conducted so far identified a number of ERP components that are altered by processing of emotional words. These are typically labeled as P1, N1, P2, Early Posterior Negativity (EPN), P3, N450, and Late Positive Complex (LPC) components (Van Hooff et al., 2008; Citron, 2012). Each of them correlates with a specific aspect of task processing or interference control required while performing the task. The first component is P1, typically observed at around 80–130 ms after stimulus onset, with the maximum located at the occipital areas (Hillyard et al., 1998; Van Hooff et al., 2008). The early timing and location suggest that P1 is the component related to early visual processing and attention employment (Citron, 2012). It has maximal amplitude over occipital regions, which suggests that it originates from the extrastriate areas of visual cortex (Sass et al., 2010). Amplitudes of P1 were found to be larger for attended than unattended stimuli (Hillyard et al., 1998). In addition valence can influence the amplitude of this component, and enlarge amplitudes for negative words compared to neutral (Van Hooff et al., 2008). The next deflection is called N1 and was found to differentiate valence of words used in a Posner-cued attention task (Pérez-Edgar and Fox, 2003). Observed amplitudes were smaller for negative than for positive and neutral words (in N1, but also in the N2 component).

The P2 component with maximum amplitude observed at about 200–250 ms (Van Hooff et al., 2008) was found in several studies to be sensitive to the emotional meaning of words. Unfortunately the pattern of results for this component is rather inconsistent. Enlarged amplitudes can be observed for positive words only (Schapkin et al., 2000), negative words only (Huang and Luo, 2006), or both positive and negative words (Carretié et al., 2004; Herbert et al., 2006). In the EST paradigm, P2 was shown to be sensitive to words related to some threat, eliciting larger amplitudes than neutral words (Thomas et al., 2007). In our previous study (Imbir et al., submitted) the P2 component was found to follow strictly the behavioral results, so it is probable that in the P2 time range the control of inhibition is manifested (Nigg, 2000). The EPN is the last one of early component associated with word processing rather than cognitive control. EPN is a negative deflection of amplitude appearing on occipito-temporal sites, peaking between 200 and 300 ms after stimulus onset (Citron, 2012). During silent reading the amplitude in EPN was found to be larger for emotionally valenced words (positive and negative) than neutral words (Kissler et al., 2007; Herbert et al., 2008). This component is therefore treated as an indicator of motivated attention.

In the literature examining ERP correlates of cognitive control, the traditional version of Stroop Task is more popular (Duncan-Johnson and Kopell, 1981; Rebai et al., 1997; West and Alain, 1999, 2000; Liotti et al., 2000; West, 2003) than the modified one, including the emotional version of this task (Metzger et al., 1997; Pérez-Edgar and Fox, 2003; Thomas et al., 2007; Van Hooff et al., 2008; Taake et al., 2009). The first component associated especially with EST performance is the N450 (West and Alain, 2000). It occurs at about 350–500 ms after stimulus onset. This component is most pronounced in fronto-central locations, but may also have a form of broadly distributed negativity (Van Hooff et al., 2008). Amplitude of this component is more negative for incongruent than congruent trials (West, 2003; West et al., 2004). The underlying mechanism might be the activation of the anterior cingulate cortex (Liotti et al., 2000). The N450 in the EST was found to be sensitive to the valence of presented words, showing greater negativity of amplitude after negative words and causing behavioral slowdown in reaction latencies (Van Hooff et al., 2008).

The second component found to be influenced by interference control in Stroop Task is P3, sometimes identified with LPC (Sass et al., 2010). The P3 in EST shows centro-posterior localization within 340–600 ms time range. This component was originally detected in the oddball paradigm and was thought to be a manifestation of surprise when less frequent stimuli appear (Luck, 2005), or it can be a manifestation of update of recalled memories content or process of event categorization (Coles et al., 2000). P3 is supposed to be reflection of automatic attention shifted to stimuli having meaning in the context of task requirements, in other words stimuli that are motivationally relevant to the task (Hajcak et al., 2010). Polarized affective valences are thought to be the factors that indicates validity of stimulation and its significance, therefore triggers attention toward such stimuli and elicits more positive amplitudes of P3 component (Naumann et al., 1992). The LPC is claimed to have a predominantly parietal distribution, peaking ~500–800 ms after stimulus onset (Citron, 2012). The amplitude is higher for threatening words than neutral ones, even without reaction latencies behavioral differentiation between categories in healthy (no trauma reported) individuals (Thomas et al., 2007). This component was found to be sensitive to valence, reward and motivational significance of the experimental procedures as well as when more controlled, explicit cognitive processes are required from the task (Citron, 2012). Some evidences suggests that LPC effects can accompany even automatic actions during execution of evaluating priming tasks (Herring et al., 2011). Nevertheless, LPC is claimed to be a manifestation of later stages of semantic processing (Sass et al., 2010; Zhang et al., 2014) associated with conscious recognition of stimulus (Hajcak et al., 2010). From that reason LPC may be interpreted as manifestation of understanding of the word connotations (Citron, 2012), but the scientific debate over this issue is still open, especially because the results for word processing in the LPC time range are rather inconsistent. Some authors (e.g., Cuthbert et al., 2000; Herbert et al., 2006, 2008) found that processing of positive words evoked a more positive LPC amplitude than neutral or negative words while others reported the opposite pattern of results (e.g., Kanske and Kotz, 2007; Hofmann et al., 2009; Schacht and Sommer, 2009; Gootjes et al., 2011); a more positive LPC amplitude to negative words than neutral or positive words. Those inconsistencies might be due to some other differences in materials used, such as concreteness (Kanske and Kotz, 2007) or the origin of affective response (Imbir, 2015; Imbir et al., 2015).

### Aim and hypothesis

The aim of our current study was to check if the factors: valence and emotional origin of stimuli modulate ERP correlates of EST processing. Valence and origin were operationalised with use of SAM scales (c.f. Figure 1 and Imbir, 2015). Using SAM scales rating we have created factorial manipulation for both factors. Current study is based on the same stimuli as our previous experiment concerning Lexical Decision Task (Imbir et al., 2016); thus it is worth to compare the results of both. The LDT involves involuntary semantic processing, but this processing does not interfere with the task performance. The EST also involves involuntary semantic processing, but this cause the interference and slowdown in reaction latencies. Results of LDT may give us chance to draw some expectations concerning origin effects in EST. Correlates of involuntarily semantic processing in LDT (Imbir et al., 2016) were localized and affected two time ranges: 290–375 and 375–670 ms after stimulus onset. The task was to discriminate words from pseudo-words; thus no meaning processing was required. First time range was identified as FN400 component, found to be a manifestation of stimuli familiarity, greater for words than non-existing pseudo-words (c.f. Curran, 2000). We found the main effect of valence in centro-frontal ROI, showing more positive amplitudes for positive words than for neutral and negative words (Imbir et al., 2016). The subsequent 375–670 ms time range we identified as LPC component and the main effect of origin was identified in left-parietal ROI. Amplitudes for Automatic and Reflective originated words were more positive than amplitudes for control words (Imbir et al., 2016).

We intended to search amplitude differences in components typically reported for EST such as P2 and N450, as well as those associated with stimuli meaning connotations and associations processing such as LPC. We expected to find the amplitude differences in P2 component to be related to behavioral differences. This expectation was based on results concerning list of words differing in arousal and subjective significance levels (Imbir et al., submitted). Another previous work indicated that automatic-originated words interfered with task performance on an EST more than reflective-originated words (Imbir and Jarymowicz, 2013). This could be due to the automatic-originated word meanings capturing attention and/or requiring more resources to suppress. If so, we might expect automatic (vs. reflective) words to elicit a larger N450 amplitude, indicating that more cognitive control resources were required to resolve the conflict induced by the triggered deviation from task demands. We might also expect reflective (vs. automatic) words to elicit a larger LPC amplitude, indicating more broad (multi-criteria based) context evaluative processes characteristic for reflective evaluative system (Jarymowicz and Imbir, 2015), that is distinct from words complexity represented in concreteness (Kanske and Kotz, 2007; Palazova et al., 2013).

## Materials and methods

### Participants

The subjects (female = 16, male = 16), aged from 19 to 26 years (*M* = 21.63, *SD* = 1.98), were students at different Warsaw colleges and universities. They took part in the experiment voluntarily, for a small reward. All of the participants were right-handed, native Polish language speakers with normal or corrected-to-normal vision. Participants provided their verbal informed consent to participate in the presence of at least two lab members, which was documented in a research diary. We did not collect any personal data from our participants, to assure their anonymity. This procedure was suggested by the bioethical committee. The design, experimental conditions and consent procedure for this study were approved by the bioethical committee of the Maria Grzegorzewska University.

### Design

We investigated the behavioral and electrophysiological measures related to the reading of emotional words. We manipulated the factors of valence (3 levels) and origin (3 levels), while controlling the following properties of words: arousal, concreteness, frequency of appearance in language and length. The distribution of variables: response accuracy and number of correct and artifact-free trials was not Gaussian, therefore the significance of effects concerning these variables was assessed by means of the Friedman test for replicated block design. The effects concerning other variables, with approximately normal distribution, were assessed by means of ANOVA with repeated measures.

### Linguistic materials

Linguistic materials were chosen from an Affective Norms for Polish Words Reload (ANPW_R: Imbir, 2016c) dataset from among 4900 Polish words. The stimuli selection was aimed to create the 3 (valence: negative, neutral and positive) × 3 (origin: automatic, not specified and reflective) factorial manipulation with control for another potentially important factors, such as arousal, concreteness, frequency, or words' length. Valence of feelings toward stimuli was measured with use of bipolar scale varied from 1(negative feelings) to 9 (positive ones). Origin scale was also bipolar and varied from 1 (of automatic origins) to 9 (of reflective origins). Only nouns from ANPW_R were selected. For the different levels of valence and origin we selected words, rated respectively: below −1 *SD*, from −0.5 to 0.5 *SD*, and above 1 *SD* from the average rating in the corresponding dimension. Further, the selected words had medium ratings (between −0.5 and 0.5 *SD*) for arousal and for concreteness. The selection procedure also ensured an equalization of the frequency of appearance and length (NoL) of words. Frequency estimations were based on online internet Polish texts (Kazojć, 2011) and represented the number of occurrences of each word in the whole database used. The distribution of values in this database was right-skewed, but was corrected by natural logarithm LN transformation enabling the application of parametric statistics. Thus, all analyses we conducted used the LN of frequency estimation. This procedure has led us to select 15 words in each of nine categories (c.f. Supplementary Material). Table 1 presents mean values of manipulated as well as controlled factors for each of 9 experimental groups of words. Table 2 presents list of words in each category.

**Table 1:** **Descriptive statistics (*M, SD*) for groups of words used in factorial manipulation (Source: Imbir et al., 2016)**.

	***M***	**(*SD*)**	***M***	**(*SD*)**	***M***	**(*SD*)**	**Origin category**	***M***	**(*SD*)**
Valence	3.50	(0.36)	5.02	(0.56)	6.71	(0.35)	Automatic	5.07	(1.39)
Origin	4.45	(0.53)	4.58	(0.37)	4.33	(0.70)		4.45	(0.55)
Arousal	4.37	(0.49)	4.15	(0.55)	4.28	(0.80)		4.27	(0.62)
Concreteness	4.31	(1.15)	3.95	(0.74)	4.48	(1.20)		4.24	(1.05)
NoL	7.20	(2.65)	7.47	(1.96)	7.40	(2.41)		7.36	(2.31)
Ln_freq	5.21	(1.91)	5.65	(2.03)	5.73	(2.28)		5.53	(2.04)
Valence	3.37	(0.36)	5.19	(0.54)	6.38	(0.32)	Control (0)	4.98	(1.32)
Origin	5.41	(0.31)	5.49	(0.30)	5.36	(0.35)		5.42	(0.32)
Arousal	4.15	(0.23)	4.12	(0.67)	4.04	(0.51)		4.11	(0.49)
Concreteness	4.05	(1.12)	3.96	(1.32)	4.17	(0.74)		4.06	(1.06)
NoL	6.47	(2.03)	5.27	(1.33)	6.93	(2.02)		6.22	(1.92)
Ln_freq	5.48	(2.28)	5.97	(1.27)	6.61	(2.02)		6.02	(1.92)
Valence	3.66	(0.35)	5.30	(0.39)	6.49	(0.40)	Reflective	5.15	(1.23)
Origin	6.46	(0.30)	6.63	(0.41)	6.63	(0.56)		6.57	(0.43)
Arousal	4.32	(0.49)	3.93	(0.47)	4.03	(0.36)		4.10	(0.46)
Concreteness	4.17	(1.13)	4.09	(1.17)	4.41	(1.07)		4.22	(1.11)
NoL	7.07	(1.75)	6.27	(1.62)	7.20	(2.27)		6.84	(1.91)
Ln_freq	5.42	(1.37)	6.53	(1.79)	6.01	(1.22)		5.99	(1.52)
**Valence category**	**Negative**		**Neutral**		**Positive**			**Total**	
Valence	3.51	(0.37)	5.17	(0.50)	6.53	(0.38)	Total	5.07	(1.31)
Origin	5.44	(0.92)	5.57	(0.92)	5.44	(1.09)		5.48	(0.97)
arousal	4.28	(0.42)	4.07	(0.56)	4.12	(0.58)		4.16	(0.53)
concreteness	4.18	(1.11)	4.00	(1.08)	4.35	(1.01)		4.18	(1.07)
NoL	6.91	(2.15)	6.33	(1.86)	7.18	(2.20)		6.81	(2.09)
Ln_freq	5.37	(1.85)	6.05	(1.72)	6.12	(1.89)		5.85	(1.84)

**Table 2:** **Full list of stimuli used in each category of experimental manipulation**.

		**Valence category**					
		**Negative**		**Neutral**		**Positive**	
Origin Category
	Automatic	*czkawka szloch łzy uszczypnięcie pijak naiwniak słabeusz zmęczenie hałas plotka grymas gafa usidlenie smarkacz zaślepienie*	hiccup sob tears pinch drunk sucker weakling fatigue noise rumor grimace blunder ensnaring stripling infatuation	*procesja kościół kuksaniec tarot loteria westchnienie jałmużna błazen mrowienie pragnienie obrzęd wróżka młodzież łasuch burza*	procession church nudge tarot lottery sigh alms clown tingling desire rite fairy youth gourmand storm	*zakochanie passa toast powitanie zapach słodycz pomoc niemowlak flirt potomstwo pozdrowienie skarb walentynka podarunek ferie*	infatuation streak toast welcome fragrance sweetness help infant flirt offspring greeting treasure valentine gift holiday
	Control (0)	*wina ciemnota truchło dół ochłap breja paszkwil kuternoga reumatyzm biedak śpiączka obtarcie błąd łachmany wada*	fault unacquaintance carcass pit offal slush libel lame rheumatism wretch coma sore error rags drawback	*doping chór kłębek telewizja guru wódka unik czara smok blef żargon głębia farsa grono pisarz*	cheering choir hank television guru vodka dodge goblet dragon bluff jargon depth farce bunch writer	*przyjęcie rejs powiew promocja klimat gość brawa kreskówka melodia wydarzenie smak południe malarstwo wyzwanie obrońca*	party cruise waft promotion climate guest applause cartoon melody event taste south painting challenge defender
	Reflective	*egzaminy ignorancja krata minus szpieg koszty podwładny podatek alimenty odsetki rząd przemyt recesja bezrobocie heretyk*	exams ignorance grating minus spy costs subordinate tax alimony interest government smuggling recession unemployment heretic	*szlachta etykieta sułtan zadatki prawo prasa stawka raport wojsko interes dyscyplina wynik weto hodowla kurs*	nobility label sultan makings right press bid report army business discipline result veto breeding course	*miliard tolerancja mistrz patent dobytek absolwent uczony stypendium szczyt równowaga oszczędności płaca satyra lider zysk*	billion tolerance master patent property graduate scholar scholarship peak balance savings wages satire leader profit

The properties of construction of the manipulation were assessed by means of 3 (valence levels) × 3 (origin levels) ANOVA analyses for each dimension measured. In the case of manipulated variables we have found for **valence ratings** significant differences for valence levels: *F*_(2, 126)_ = 607.44, *p* < 0.001, η^2^ = 0.91, but not for origin levels: *F*_(2, 126)_ = 1.88, *p* = 0.16, η^2^ = 0.03, nor for interaction between valence and origin levels: *F*_(4, 126)_ = 2.09, *p* = 0.086, η^2^ = 0.062. For **origin ratings** we have found significant differences for origin levels: *F*_(2, 126)_ = 254.55, *p* < 0.001, η^2^ = 0.80, but not for valence levels: *F*_(2, 126)_ = 1.27, *p* = 0.28, η^2^ = 0.02, nor for interaction between valence and origin levels: *F*_(4, 126)_ = 0.5, *p* = 0.74, η^2^ = 0.016.

In the case of controlled variables no statistically significant effects were found for three dimensions. **Arousal ratings**: no statistically significant effects were found [statistics summary: between valence levels: *F*_(2, 126)_ = 1.98, *p* = 0.14, η^2^ = 0.02, origin levels: *F*_(2, 126)_ = 1.44, *p* = 0.24, η^2^ = 0.02, interaction between valence and origin levels: *F*_(4, 126)_ = 0.5, *p* = 0.72, η^2^ = 0.016]. **Concreteness ratings**: no statistically significant effects were found [statistics summary: for valence levels: *F*_(2, 126)_ = 1.19, *p* = 0.31, η^2^ = 0.02, for origin levels: *F*_(2, 126)_ = 0.4, *p* = 0.67, η^2^ = 0.006, for interaction between valence and origin levels: *F*_(4, 126)_ = 0.12, *p* = 0.98, η^2^ = 0.004]. **Frequency of the words' appearance** in the Polish language (after logarithm transformation, with data taken from Kazojć, 2011, dataset), showed no statistically significant effects [for valence levels: *F*_(2, 126)_ = 2.3, *p* = 0.11, η^2^ = 0.04, for origin levels: *F*_(2, 126)_ = 1.0, *p* = 0.37, η^2^ = 0.016, for interaction between valence and origin levels: *F*_(4, 126)_ = 0.44, *p* = 0.78, η^2^ = 0.014]. **Average length of the words** revealed no significant effects either between valence levels [*F*_(2, 126)_ = 2.01, *p* = 0.14, η^2^ = 0.03], or the interaction between valence and origin groups of levels [*F*_(4, 126)_ = 0.82, *p* = 0.52, η^2^ = 0.025]. But there was a difference between origin levels [*F*_(2, 126)_ = 3.48, *p* = 0.034, η^2^ = 0.052]. The *post-hoc* analysis showed that the difference concerned words of an automatic origin vs. words of no particular origin: *t*_(132)_ = 2.62, *p* = 0.01. Words of automatic origin were *M* = 7.3 (*SEM* = 0.3) letters long while words of no particular origin were *M* = 6.2 (*SEM* = 0.3) letters long. The other differences appeared insignificant. The linguistic materials are the same as in our previous studies (e.g., Imbir et al., 2016); thus more details concerning linguistic materials properties can be obtained there (c.f. Table 1, Imbir et al., 2016). A full list of stimuli used in the experiment and their affective assessments values is presented in Appendix 1 (Supplementary Material).

### Procedure

Subjects were seated in a comfortable chair. The words were displayed on a 15.6-inch LCD screen at a distance of ~1 m from the subjects' eyes. The font was Helvetica 50 point size. Simultaneously with the target word cues indicating initial letters of Polish names of possible colors: P—orange (*pomarańczowy*), C—red (*czerwony*), Z—green (*zielony*), N—blue (*niebieski*), was displayed at the bottom of the screen. Each participant performed a training session to learn what the task was and how to perform it correctly. The training consisted of 20 initial trials (naming color squares displayed in one of the four target colors, reading color-meaning words) followed by 60 standard Stroop Tests (Stroop, 1935) i.e., naming the font color—both congruent and incongruent presented in random order. After those trial sessions, the main experiment was introduced, based on EST with use of emotional words selected. Each time participants were encouraged to respond as quickly and as accurately as possible. The subject's task in the main part of the experiment was to indicate the font color of the emotional words by pressing a response key labeled by the one of the letters P, C, Z, N. The experimental protocol is depicted in Figure 2.

The timing of a single trial in the main part of experiment was the following: a fixation cross was displayed for 700 ms; next a word was presented for as long as it took the subject to read and respond to it (no timeout was implemented, the exceptionally long responses were excluded from the offline analysis); after detecting the response the screen went blank for 300–400 ms. The trials were grouped, so that 15 words of homogeneous properties (i.e., the same level of valence and origin) were presented consecutively. We decided on a block design because EST effects are more pronounced in this type of presentation, in fact larger behavioral effects were found for block design in comparison to fully random presentation of words (c.f. Bar-Haim et al., 2007). The subject could rest for 3 s after the presentation of each group. There were altogether nine groups, one for each possible combination of factor levels (3 valence × 3 origin), comprising a list of 9 groups (9 × 15 = 135 words). The order of groups on the list, as well as the order and font color of words within each group, was fully randomized for each participant in each repetition. The experimental session had three repetitions of the list separated by a longer, self-adjusted by the subject, break. This means that single group of words (e.g., negative of reflective origins) consisted of 45 trials (3 × 15). When considering main effects, single group of words (e.g., automatic originated) consisted of 135 trials (3 × 45).

The whole experiment was composed of three repetitions of 2 distinct lists of words. First list (described above) was designed to measure valence-origin factors influence on EST. Second list was designed to operationalize factorial manipulation of arousal and subjective significance, the two activational factors postulated in dual-mind model of Emotion-Cognition interactions (Imbir, 2016b). The second list was the same as used in earlier behavioral study with dual-mind approach to understanding of EST (c.f. Imbir, 2016a). One hundred and thirty-five items from the second list contrasted orthogonally 3 levels of arousal (low, medium, high) and 3 levels of subjective significance (low, medium, high). Also valence, concreteness, frequency of appearance and length were controlled. The tasks, related to each list did not interfere with each other because they were separated in time, due to block design used for stimuli presentation. The order of lists presentations was randomized between subjects.

### EEG material

#### Apparatus

Stimuli were displayed on a 15.6-inch LCD display controlled by a PC. A second PC was used for recording EEG data. Stimuli and EEG data were synchronized using a custom-made hardware trigger. The trigger consisted of a light sensor measuring the brightness of a small rectangular portion of the screen, which was covered by the sensor. Brightness of that part of the screen was modulated simultaneously with the stimulus presentation. The signal from the sensor was recorded, together with the EEG signal, on an auxiliary input of the amplifier. This auxiliary signal was later used to align trials. EEG activity was recorded from 19 derivations of 10–20 system: Fz, Cz, Pz, Fp1/2, F7/8, F3/4, T3/4, C3/4, T5/6, P3/4, O1/2, referenced to linked earlobes, grounded on the clavicle. The impedances of electrodes were below 5 kΩ. The signal was acquired using a Porti7 (TMSI) amplifier at 256 Hz sampling frequency.

#### Offline EEG signal processing

The offline processing of the signal was performed in Matlab® with the EEGLAB (Delorme and Makeig, 2004) toolbox. The signal was zero-phase filtered with Butterworth high- and low-pass filters (2nd order, corresponding to 12 dB/octave roll-off, with half amplitude cut-off frequency = 0.1 Hz and 30 Hz respectively), and with an IIR notch filter at 50 Hz, to remove line noise. Epochs from −200 ms pre-stimulus to 850 ms post-stimulus were extracted and baseline-corrected (baseline data taken from −200 to 0 ms).

The statistical tests were implemented using the appropriate R procedures (R Development Core Team, 2008, available from http://www.R-project.org). Trials with erroneous responses, or corrupted with artifacts (e.g., eye blinks or muscle activity), or with extremely short (shorter than 2.5 percentile of the distribution of all response latencies), or long (longer than 97.5 percentile of the distribution) response latencies were excluded from the ERP analysis. The mean number of trials remaining in each of the 9 manipulation condition (from the initial 45) was *M* = 37 (*SEM* = 0.3). The Friedman test for replicated block design did not indicate significant differences in the average number of trials per condition for the origin groups with valence as a blocking variable [$\chi_{\left(2\right)}^{2}$ = 3.4, *p* = 0.2], nor for the valence groups with origin as a blocking variable [$\chi_{\left(2\right)}^{2}$ = 0.46, *p* = 0.8].

## Results

### Behavioral measures

The mean response accuracy was *M* = 90% (*SEM* = 0.4). The Friedman test for replicated block design did not indicate significant differences in the average accuracy per condition for the valence groups with origin as a blocking variable [χ_(2)_ = 1.08, *p* = 0.58], or for the origin groups with valence as a blocking variable [χ_(2)_ = 5.88, *p* = 0.053]. The response latency was analyzed for the trials that were accepted for ERP analysis (i.e., artifact free, correct responses, without the 5% most extreme RT values). Analysis by means of 3(valence levels) × 3(origin levels) ANOVA with repeated measures applied to log transformed reaction latencies did not reveal any significant effects [factor valence: *F*_(2, 62)_ = 0.84, *p* = 0.44; factor origin: *F*_(2, 62)_ = 0.65, *p* = 0.53; valence × origin interaction: *F*_(4, 124)_ = 2.0, *p* = 0.1]. The average response latency was *M* = 820 (*SEM* = 8.6) ms.

### Electrophysiological data

#### Selection of time windows and regions of interest

**Figure 3:**
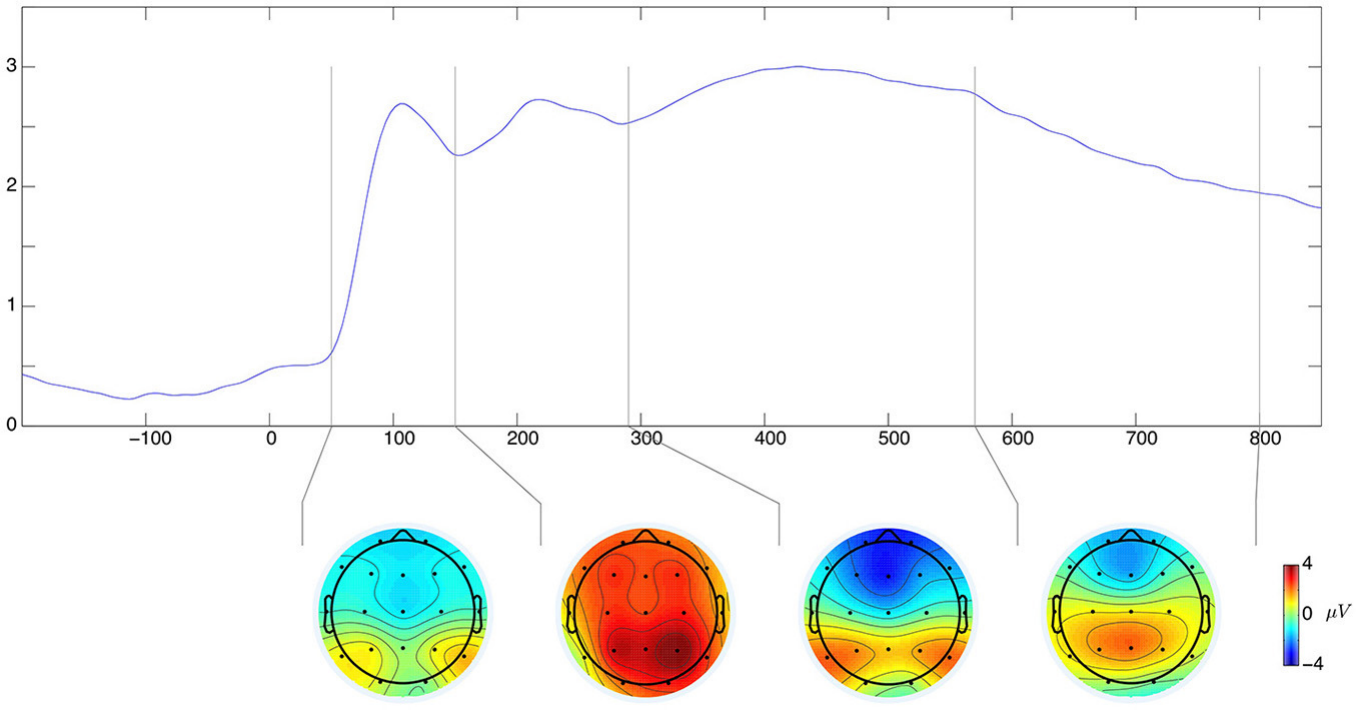
**Global field power with the selected time-windows marked by vertical lines**. Below–average amplitude topographies for each of the windows.

The following time windows were selected for evaluation of ERP effects: 50–150, 150–290, 290–570, 570–800 ms. This selection is based on the global field power curve GFP (Figure 3). The GFP is evaluated as spatial standard deviation. It quantifies the sum of electrical activity over all electrodes at a given time point. The latencies of GFP maxima may be interpreted as the latencies of evoked potential components (Lehmann and Skrandies, 1980; Skrandies, 1990). Since we do not expect lateralization effects, three regions of interest (ROI) were selected as follows: frontal (F) (electrodes: F3, Fz, F4), central (C) (electrodes: C3, Cz, C4), and parietal (P) (electrodes: P3, Pz, P4). A similar approach can be found in the other studies focusing on neural correlates of EST task (e.g., Schirmer and Kotz, 2003; Thomas et al., 2007; Taake et al., 2009) and gives us a chance to investigate a distribution of effects in a front-to-back dimension.

### Analysis of ERP effects

**Figure 4:**
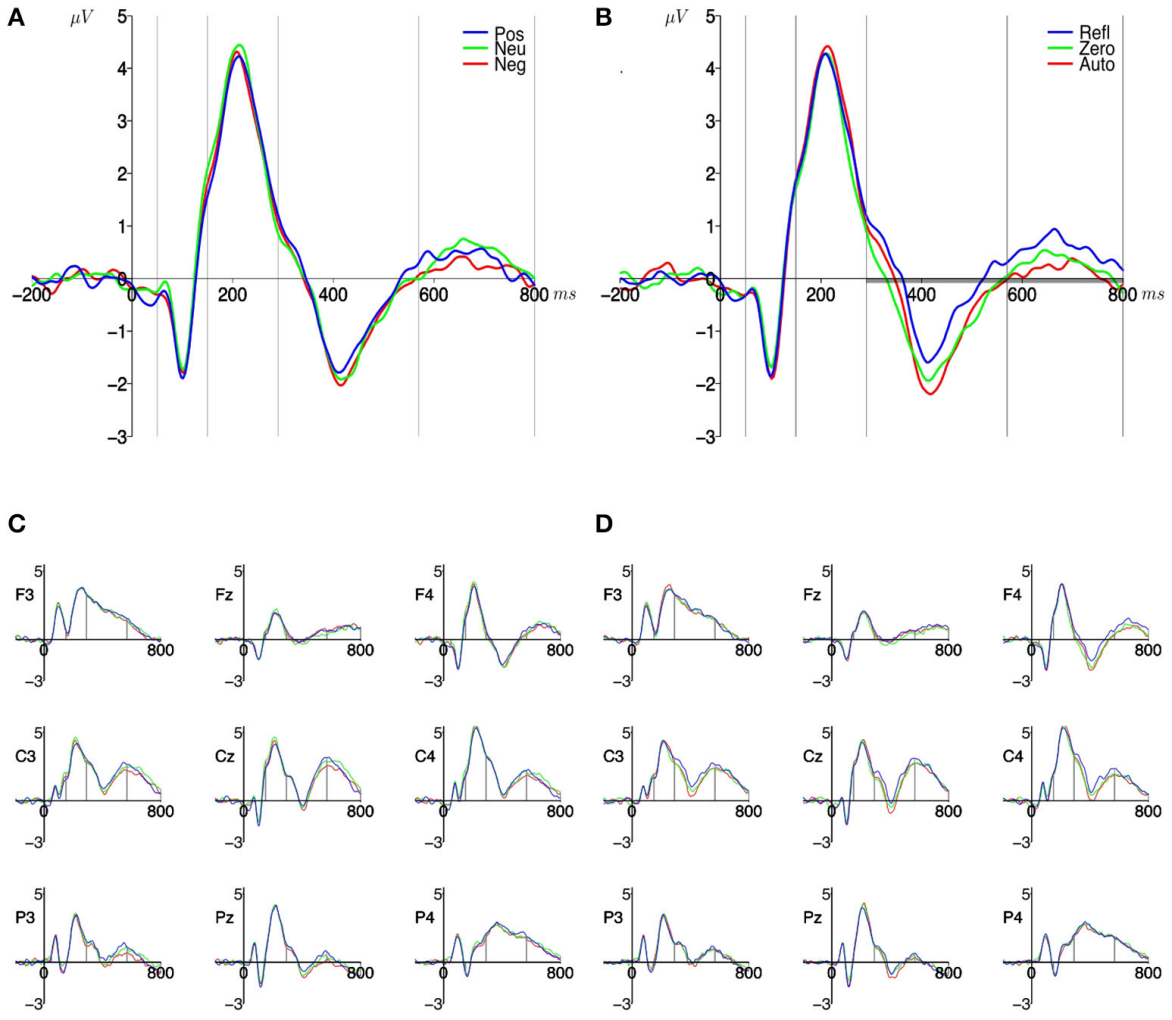
**Grand mean ERPs for the three levels of: (A) valence dimesion, (B) origin dimension, averaged across the three ROIs**. The thick gray horizontal lines indicate the time-windows with significant differences between the origin levels. Signal from each electrode included in ROIs is presented respectively below: **(C)** for valence and **(D)** origin. The symbols in legend are the labels of valence levels (Pos, positive; Neu, neutral; Neg, negative), and origin levels (Refl, reflective; Zero, of no specific origin; Auto, automatic). Horizontal axis–time in ms, vertical axis–amplitude in μV. Vertical gray lines mark the edges of the selected time-windows.

The analysis was performed by applying a three-factor repeated measure analysis of variance (origin × valence × ROI) to the mean amplitude from each subject, in each of the time windows. For each of the time windows there was a significant main effect of ROI but this finding is not interesting and will not be discussed further. There were no significant interaction between ROI and the other two variables in any of the time windows, therefore further on the amplitudes averaged across ROIs were analyzed. No statistically significant effects were observed for time windows 50–150, 150–290 ms. No effects were obtained for valence levels in any time window (Figure 4A). But for two time windows there were significant effects for origin levels. Namely, in time window **290–570 ms** a main effect of origin [*F*_(2, 62)_ = 5.078, *p* < 0.01] was obtained. The amplitude was less negative for stimuli with reflective origin (*M* = −0.27, *SEM* = 0.33) than for those with automatic origin (*M* = −0.72, *SEM* = 0.30), and with no specific origin (*M* = −0.81, *SEM* = 0.30); corresponding *t*-test results [*t*_(31)_ = 2.37, *p* < 0.05; *t*_(31)_ = 2.83, *p* < 0.02]. In the time window **570–800 ms** a main effect of origin [*F*_(2, 62)_ = 4.78, *p* < 0.016] was obtained too, but the pattern of differences was slightly different. Only the amplitude for reflective stimuli (*M* = 0.56, *SEM* = 0.27) was more positive than for automatic origin (*M* = 0.13, *SEM* = 0.26); corresponding *t*-test result [*t*_(31)_ = 2.92, *p* < 0.02]. No statistically significant effects of interaction between valence and origin were observed in these time windows [**290–570 ms:** *F*_(4, 124)_ = 1.37, *p* = 0.25; **570–800 ms:** *F*_(4, 124)_ = 0.46, *p* = 0.77]. The time course of the ERPs, which illustrate the results, is shown in Figure 4B. Since no significant interactions between origin levels and ROI were observed, the curves in Figure 4 are the amplitudes of ERP averaged across all three ROIs.

## Discussion

This study was focused on investigating the role of origin and valence of emotional words in the processing of EST and involuntary word processing. We hoped to find electrophysiological correlates of underlying mechanisms, even when the behavioral outcomes of the task are not visible (c.f. Thomas et al., 2007). Result confirmed that there are no differences in response latencies; meanwhile ERP amplitude differs between levels of origin of emotion included in word meaning, but not between valence levels. The differences appeared in time windows characteristic to components thought to be manifestations of cognitive control (N450: **290–570 ms)** and involuntary word processing including more controlled and explicit cognitive processing of words meaning (LPC: **570–800 ms**). Surprisingly, electrophysiological results were not localized at a particular site, but rather generally distributed over all the ROIs analyzed.

### Behavioral results

As expected, we did not find behavioral differences in reaction latencies due to the type of word processed in EST. Those results are coherent with our other unpublished negative results concerning behavioral-only measures for the same list of words as used now. This lack of difference is also consistent with EEG measures in the P2 component, found in our earlier study (Imbir et al., submitted) to be a strict correlate of behavioral differences. Lack of behavioral differences related to variations of valence dimension can be attributed to the role of another dimensions found to be more crucial (c.f. Burt, 2002). Previous behavioral findings showed that arousal (Dresler et al., 2009) or frequency of appearance (Burt, 2002) or origin (Imbir and Jarymowicz, 2013) could account for effects shown in early studies with EST (c.f. Burt, 2002; Larsen et al., 2006; Imbir, 2016a). This is also consistent with other EEG studies showing no behavioral differences (c.f. Thomas et al., 2007) for valence, while reporting ERP amplitude differences.

In this study, we did not find the results for the origin of the affective state, reported in our earlier studies involving EST (c.f. Imbir and Jarymowicz, 2013), where slowdown was caused by automatic originated stimuli, but not by reflective originated or neutral ones (with no effects of valence of emotion). The most probable reason for such difference in the results of both studies may be the method of selection of stimuli for both experiments. Imbir and Jarymowicz (2013) based the selection on judge-competent decisions of compliance with the automatic and reflective origins definitions. This makes stimuli explicitly connected with automatic or reflective origins, but not controlled for arousal or concreteness. In addition the content of the words was specified as labels of characteristic for both origins' emotional states or objects causing those states. This mean that participant could explicitly find out the actual aim of experiment. Our current study is based on a more precise stimuli selection from a large amount of words, with properties checked in advance by a different group of participants (c.f. Imbir, 2015, 2016a,c). For that reason, the choice of stimuli could not be explicitly attributed to certain origin by a participant, but the context of a whole list made sense of a specific category meaning. Another difference is precise control for arousal, concreteness, and frequency differences in stimuli presented in this current study (c.f. Supplementary Material). It is possible, that the behavioral results shown in EST were caused mostly by these dimensions (Burt, 2002; Larsen et al., 2006; Thomas et al., 2007), and not by valence or origin themselves. This could mean, that the interpretation of EEG results should be made more in the context of involuntary word processing, than cognitive control measured in EST. Nevertheless, lack of behavioral results does not exclude the urge for searching electrophysiological correlates of processing in the EST experiment (Thomas et al., 2007).

### ERP results

Electrophysiological correlates of EST allowed us to inspect the stages of task processing and the role of valence and origin dimensions of words used in this process. What is the most interesting is that we found no effects of valence during the whole time course analyzed. It seems that if the origin of an affective state is controlled and aligned in all valence conditions, the traditional effects may disappear (Imbir and Jarymowicz, 2013). We claim that origin is one of the properties of affective reaction (Imbir, 2015) that can be attributed to distinct mind systems underlying formation of this reaction (Gawronski and Creighton, 2013; Jarymowicz and Imbir, 2015). Since affective reaction can be described in bimodal affective space of valence and arousal (Russell, 2003), mostly arousal differences were claimed to cause EST effect on both behavioral (Burt, 2002; Larsen et al., 2006; Dresler et al., 2009; Imbir, 2016a) and electrophysiological levels (Metzger et al., 1997; Pérez-Edgar and Fox, 2003; Thomas et al., 2007; Van Hooff et al., 2008; Taake et al., 2009; Imbir et al., submitted).

The lack of amplitude differences in P2 time range observed in this study corresponds with the lack of behavioral differences in response latencies. Previously mentioned second list used in this experimental protocol [contrasting 3 levels of arousal and 3 levels of subjective significance (Imbir et al., submitted)] resulted in both reaction latencies differences due to both factors as well as amplitude differences in P2 (150–290 ms) component closely resembled the pattern of behavioral results (longer reaction times ~ more positive amplitude). Thomas et al. (2007) suggested that P2 amplitude might be a more sensitive measure of inhibitory control than behavioral responses. Valence and origin list of words results reported in current paper supports this claim, the same as result for arousal and significance list of words (Imbir et al., submitted).

The results concerning origin of emotional state consequences for EST processing (c.f. Figure 4B) are interesting. We found global effects in two time ranges that can be attributed to the N450 and LPC components. The first component, peaking at about 350–500 ms after stimulus onset, called N450 (c.f. West and Alain, 2000) is frequently reported (Van Hooff et al., 2008; Taake et al., 2009) in studies with EST paradigm. The N450 is located in the frontal regions of a head (Sass et al., 2010). Although no interaction with ROI was found for amplitudes in this study, the topography of the average amplitude distribution for this time window (c.f. Figure 3, bottom graphs) suggest that the most intensive negativity is indeed located in the frontal regions of the head. The amplitude of the N450 component was found to be more negative for incongruent than congruent trials (West, 2003; West et al., 2004). The underlying mechanism is possibly associated with conflict detection (West, 2003; West et al., 2004) or selection of competing responses (West and Alain, 1999). In ERP waveform we can see the N450 component (c.f. Figure 4B, 290–570 ms). The reflective originated conditions generated less negative amplitude than automatic originated and control words. This mean, that less incongruent were the reflective originated stimuli presentation conditions, thus one may expect that indeed the rational mind reduces interference of automated reading and meaning of stimuli understanding, even without behavioral outcomes in response latencies. The lack of correspondence with behavioral results may suggest, that decision concerning the type of answer is made earlier (in P2 time range), while N450 in this study reflect rather the conflict cost appearing after decision was made. Alternatively, they may be interpreted in the context of involuntary processing of a meaning of words included in manipulations (see below).

The last component, the LPC, is a manifestation of explicit and controlled cognitive activation of the content and connotations of a word, but rather made without explicit instruction in the context of current experiment. This is a late word-processing related activity rather than a cognitive control manifestation, especially we may assume that this a kind of post-semantic processing (Jończyk, 2016) of stimulus meaning. At the LPC time range some specific differentiation between distinct valences (Zhang et al., 2014) is manifested, but the debate over the nature of emotional valence still remains open question. We may claim that origin of an affective state is some kind of emotional complexity, derived from underlying mechanisms proposed by the duality of mind approach, but clearly distinct from concreteness precisely controlled in our studies (Imbir et al., 2016, submitted). The stimuli differing in complexity have to be processed in a different way when semantic meaning is considered; thus differences in LPC are expected and probable. In the literature (for review see: Citron, 2012), LPC is claimed to be sensitive to valence differences, but up to this point the emotional complexity of valenced stimuli has not been the subject of special attention of the scientific community. We mentioned in the Introduction Section that the LPC results were rather inconsistent; thus repetition of origin effects with no valence-related differences may suggest that origin is a more salient factor for this stage of processing (c.f. Imbir et al., 2015, 2016; Imbir et al., submitted). An interesting insight into this problem may be given by studies focusing on consequences of the concreteness dimension for word processing, done mostly in the LDT paradigm (e.g., Kanske and Kotz, 2007; Palazova et al., 2013). They showed that abstract words (both nouns and verbs) elicited more positive LPC amplitude responses than concrete nouns and verbs. This is in line with current results showing that reflective (more emotionally complex) originated words elicited more positive amplitude than automatic originated (less emotionally complex) even when both groups are matched for concreteness (cognitive complexity). Our analyses of words used in this experiment (c.f. Imbir et al., 2016) showed that origin and concreteness dimensions share no more than 10% common variance when all 4,905 words from ANPW_R (Imbir, 2016c) are considered.

It is worth highlighting that results of our study were not localized in a specific site or ROI. This is a different result to other studies showing localized effects. It may be partially due to the half-exploratory analytical strategy chosen in this study. When ANOVA showed no significant interaction between one of the main factors and the ROI factor, we simply resigned from further investigation inside ROIs, despite the fact, that average amplitude distribution showed some topographical variations (c.f. Figure 3). In fact, effects found are distributed in the same way all over the head. This strategy allows us not to miss potentially important findings connected with new proposed origin dimension. Although some results with origin exist so far (c.f. Imbir et al., 2015, 2016; Imbir et al., submitted), the differences in tasks used still make us careful about potentially unpredicted effects.

It is worth to compare the results of current study to the results of study with use of LDT paradigm applied for the same list of stimuli (c.f. Imbir et al., 2016). Both EST and LDT tasks have in common the fact that processing of words' meaning is not required by the task; thus rather implicit and involuntary. In LDT participants have to react to stimuli type, while in EST, the task is to ignore the stimulus at lexical level; thus both are different to some extent. Comparing the results of this current study to those concerning LDT, one can see that no valence effects are visible in LPC, but origin differences look different in both paradigms. The difference concerned Automatic originated words having similar amplitude to Reflective originated words in LDT, while being lower in EST paradigms. We argue that Reflective originated stimuli should activate the resources for the controlled part of EST (c.f. Introduction Section; Imbir, 2016a); thus cortical response to them should be larger and should elicit more positive LPC. It is likely that in LDT, the origin construct has nothing in common with performance in the task; thus both origins generated similar amplitudes. The differences found earlier were associated with different components, specific to the used tasks; thus are hard to compare straightforward. In LDT the valence factor was present in the results pattern, while in EST no valence effects were found. We may assume that valence is the intuitive dimension on a subjective level (Russell, 2003), but probably not as important as one may expect in EST phenomenon.

Finally we would like to note, that in current study we have used a relatively low number of stimuli repeated three times. This is a result of a compromise between careful selection of stimuli and balancing them in all controlled dimensions, and the need to have enough events for averaging ERPs. In fact, the repetition of stimuli may potentially lead to attenuation of behavioral and ERP effects. Lack of valence related differences observed in results might be therefore attributed to this methodological issue. However, the fact that in these circumstances we observe an effect of origin supports the postulated higher importance of origin dimension even stronger.

## Conclusion

Results of this study showed that despite the lack of behavioral results, the processing of EST causes differences in electrophysiological correlates of this task. We have demonstrated that there were no differences in the P2 component, found in another study (Imbir et al., submitted) to be a strict manifestation of behavioral differences. We also found only origin, but not valence, shaped cortical responses of the brain while processing words in EST. It is possible that without including origin factor in experimental schema valence differences can be detected. When origin is controlled, the differences in amplitudes for negative as well as positive words can disappear.

## Authors contributions

All authors contributed to final version of the manuscript. Theoretical proposition: KI; Design: KI, JŻ Method (words): KI; Method (EEG measures) JŻ, TS; Experimental procedure programming: TS, JŻ Experiment execution: TS, JŻ Statistical analyses: JŻ, KI, TS, JD, GB; Results description: JŻ, JD; Results discussion: KI; Figures: JŻ, TS, KI, JD, GB.

### Conflict of interest statement

The authors declare that the research was conducted in the absence of any commercial or financial relationships that could be construed as a potential conflict of interest.
